# Analysis of Heterosis and Quantitative Trait Loci for Kernel Shape Related Traits Using Triple Testcross Population in Maize

**DOI:** 10.1371/journal.pone.0124779

**Published:** 2015-04-28

**Authors:** Lu Jiang, Min Ge, Han Zhao, Tifu Zhang

**Affiliations:** 1 Provincial Key Laboratory of Agrobiology, Jiangsu Academy of Agricultural Sciences, Nanjing, China; 2 School of Biosciences, University of Nottingham, Sutton Bonington, United Kingdom; China Agricultural University, CHINA

## Abstract

Kernel shape related traits (KSRTs) have been shown to have important influences on grain yield. The previous studies that emphasize kernel length (KL) and kernel width (KW) lack a comprehensive evaluation of characters affecting kernel shape. In this study, materials of the basic generations (B73, Mo17, and B73 × Mo17), 82 intermated B73 × Mo17 (IBM) individuals, and the corresponding triple testcross (TTC) populations were used to evaluate heterosis, investigate correlations, and characterize the quantitative trait loci (QTL) for six KSRTs: KL, KW, length to width ratio (LWR), perimeter length (PL), kernel area (KA), and circularity (CS). The results showed that the mid-parent heterosis (MPH) for most of the KSRTs was moderate. The performance of KL, KW, PL, and KA exhibited significant positive correlation with heterozygosity but their Pearson’s R values were low. Among KSRTs, the strongest significant correlation was found between PL and KA with R values was up to 0.964. In addition, KW, PL, KA, and CS were shown to be significant positive correlation with 100-kernel weight (HKW). 28 QTLs were detected for KSRTs in which nine were augmented additive, 13 were augmented dominant, and six were dominance × additive epistatic. The contribution of a single QTL to total phenotypic variation ranged from 2.1% to 32.9%. Furthermore, 19 additive × additive digenic epistatic interactions were detected for all KSRTs with the highest total *R^2^* for KW (78.8%), and nine dominance × dominance digenic epistatic interactions detected for KL, LWR, and CS with the highest total *R^2^* (55.3%). Among significant digenic interactions, most occurred between genomic regions not mapped with main-effect QTLs. These findings display the complexity of the genetic basis for KSRTs and enhance our understanding on heterosis of KSRTs from the quantitative genetic perspective.

## Introduction

Heterosis was proposed in the early 20^th^ century to describe the superiority of heterozygous F_1_ compared with its homozygous parents in one or more traits [1,2]. Since then, heterosis has been widely applied for improving crops, and it has been particularly effective for maize production [3–5]. In general, maize crossbreeding efforts first aimed at improving the inbred lines and subsequently hybridizing these lines. Usually, the methods were focused on improving grain yield, which directly affects corn production and/or characters indirectly acting on corn production, e.g., decreasing plant height [6,7], enhancing resistance to diseases and pests [8–10], increasing planting density [11–13], or enhancing fertilizer utilization efficiency [14–16].

Given the quantitative complexity of these studies, grain yield was usually dissected into several components for further analysis. Considered the morphological relations, the relative components could be divided into two parts, ear related traits (e.g., ear length, ear diameter, row numbers, kernel number per row, and kernel number per ear) and kernel related traits (e.g., kernel length (KL), kernel width (KW), kernel thickness, and kernel weight). Researchers have demonstrated that yield related components always exhibit higher heritability than grain yield [17]. Most previous studies on maize yield related traits focused on ear related traits [18–21]. In recent years, kernel related traits have garnered more attention with studies attempting to elucidate the genetic basis of grain yield for a variety of reasons. For example, kernel size and weight were characterized as important determinants of grain yield [22,23] and large inbred kernels had the potential to produce better early vigor hybrids and promote flowering time [24]. In addition, several reports revealed that KL and KW had strong influences on kernel weight [25,26]. Therefore, kernel shape related traits (KSRTs) such as KL and KW are likely the major characters affecting grain yield.

Analyses based on quantitative trait locus (QTL) mapping have been extensively applied for deciphering the genetic basis of kernel shape in major crops [27–34]. In contrast, the corresponding research progress in maize has been slow and only a few QTLs related to kernel shape have been detected [17,26,35,36]. However, these studies all focused on the relationships of kernel weight with KL and/or KW using different mapping populations, e.g., F_2:3_ and recombinant inbred line (RIL). To date, there have been no consistent QTLs related to KL and KW found among previous reports. The discrepancy could be caused by the different evaluation methods, different linkage maps, or different mapping populations used. In addition to KL and KW, other kernel shape characters such as perimeter length (PL), kernel area (KA), and circularity (CS) have not be quantified in previous studies on maize.

The accurate estimation of genetic effects facilitates a better understanding of target traits. To precisely detect epistasis, the triple testcross (TTC) design was developed by Kearsey and Jinks [37]. The design has the ability to test epistasis with high efficiency and can produce unbiased estimates of additive and dominance effects if epistasis does not exist. Following the RIL-based TTC design, digenic epistatic effects have been evaluated in several studies [38–40]. In maize, Frascaroli et al. [41] mapped several QTLs for plant height, seedling weight, grain yield, and number of kernels per plant using a TTC design and identified a few QTLs for these traits with digenic epistasis.

In the present study, the software *SmartGrain* was used for comprehensively and precisely evaluating characters affecting kernel shape: KL, KW, length to width ratio (LWR), PL, KA, and CS [42]. The TTC populations were created to test for additive, dominant, and epistatic effects. The main objectives of this work were to: (1) evaluate the level of heterosis for KSRTs; (2) investigate the correlations between KSRTs,heterozygosity, and yield; (3) estimate the number, genomic position, and the genetic effect of QTLs related to KSRTs for kernel shape; (4) characterize the mode of gene action for these QTLs; and (5) detect any digenic epistatic effects and their contributions to phenotypic values for KSRTs.

## Materials and Methods

### Plant materials

The intermated B73 × Mo17 (IBM) populations derived from the B73 × Mo17 cross were used for creating the TTC genetic populations. Based on the four generations of intermating among the F_2_ progenies, the IBM populations have the potential to increase the genetic resolution roughly four-fold [43]. According to the testcross (TC) progeny production scheme [37], three TC populations were obtained as follows: three groups of 82 RILs (female parents) were crossed with B73, Mo17, and their F_1_ (B73 × Mo17) and the corresponding progeny populations are referred to as TC(B), TC(M), and TC(F). Therefore, the materials tested included the basic generations consisting of B73, Mo17, and F_1_ as well as four RILs, TC(B), TC(M), and TC(F) populations, with 82 genotypes within each population.

### Field experiments

All materials were planted in three blocks in the Luhe Experimental Station of the Jiangsu Academy of Agricultural Science. Each block was arranged with the basic generations and the four populations. To evaluate the population traits statistically, two different field designs were laid out. A randomized complete block design was used for the basic generations and a split-plot design was used for the RIL and TTC populations. In the split-plot design, the four populations were deemed the main plot and the subplot was comprised of the RILs and their three TTC progenies. In all instances, each genotype within each block was planted in a single-row with 8 plants after thinning. Thus, the distance between plants in a row was 0.3 m and the distance between rows was 0.6 m. To reduce the boundary effect of the field, border rows were grown all around the blocks. All materials were subjected to the normal field management until the harvest of mature ears.

### Traits measurement

The whole ears were manually harvested at physiological maturity (R6 stage). After several days of airing, the middle part of each ear was shelled. All kernels in a row were then bulked and 30 kernels were selected randomly per bulk to survey the representative KSRTs of genotypes. To measure the kernel shape accurately, *SmartGrain*, a high-throughput software for seed shape measurement was employed for the analysis [42]. Following the manual, digital images of thirty kernels per genotype (along with a wire for reference length) were captured by large pixel digital camera. The images were then analyzed and kernel outlines were detected by *SmartGrain*. The software calculated six KSRTs automatically including KL, KW, LWR, PL, KA, and CS. We corrected misdetected parameters manually. Furthermore, 100-kernel weight (HKW) was evaluated by averaging three replicates of randomly selected 100 kernels for each TTC population.

### Genetic linkage map

The release of B73 reference genomic data (V2) and Mo17 genomic sequencing data facilitates development of novel molecular markers. Based on these genomic sequencing data, 93 insertion/deletion (InDel) and 57 presence/absence variation (PAV) polymorphisms exploited between B73 and Mo17 were used for genotyping 302 IBM RILs in our approach (S1 Table). This was also done with 142 single nucleotide polymorphisms (SNP) called based on the RNA-based sequencing data of IBM RILs deposited in NCBI (SRA054779)[44]. Moreover, 667 public markers with genotyping information were selected randomly to incorporate into the genotyping data. In total, 959 molecular markers were used for the genetic linkage map construction by MSTMap (S2 Table) [45].

### Data analysis and QTL mapping

Heterosis was evaluated by mid-parent heterosis (MPH): MPH (%) = (F_1_-MP)/MP × 100, where MP is the mid-parent value. The performance of the cross progenies TC(B), TC(M), and TC(F) is denoted as *L*_*1i*_, *L*_*2i*_, and *L*_*3i*_ (*i* = 1–82), respectively. The mean values of genotype were used for analyses of significant difference which were conducted via the SPSS 17.0 software (http://www.spss.com). As described by Melchinger et al. [46], the linear transformations *Z*_*1*_ = (*L*_*1i*_ + *L*_*2i*_)/2, *Z*_*2*_ = *L*_*1i*_ - *L*_*2i*_, and *Z*_*3*_ = *L*_*1i*_ + *L*_*2i*_ - 2*L*_*3i*_ were then calculated for additive, dominance, and epistasis QTL analysis.

The QTL analysis was carried out on the three linear transformations *Z*_*1*_, *Z*_*2*_, and *Z*_*3*_ with composite interval mapping [47,48] via the software QTLMapper [49]. To obtain high resolution QTL mapping, the main and interaction effects of important markers were taken as the background genetic variation control. In the mapping range setup, the whole genome range was chosen to search QTLs from the entire genome. Following the manual, the main-effect QTLs were first identified and then the digenic epistasis at all possible marker pairs was analyzed. When the threshold of *P*≤0.001 and *R* ^*2*^>5% was reached, the putative QTL was declared present [49]. If *P*≤0.001 but 0<*R* ^*2*^≤5%, QTLs with LOD>3.0 were also considered significant. For all the main-effect and epistasis QTLs, the detection was based on the mix model approach [49].

Regarding the genetic effects of QTLs, the TTC design revealed the novel definitions given by Melchinger et al.[46] as follows. QTL detected by one-dimensional genome scans with *Z*_*1*_, *Z*_*2*_, and *Z*_*3*_ reflect augmented additive effects *a*_*i*_ *, augmented dominance effects *d*_*i*_ *, and dominance × additive effects [*da*_*i*_], respectively. The dominance degree ratio of a QTL was estimated by the augmented dominance ratio |*d*_*i*_ */a_*i*_ *| and the QTLs were classified as additive (|*d*_*i*_ */a_*i*_ *|<0.2), partially dominant (0.2≤|*d*_*i*_ */a_*i*_ *|<0.8), dominant (0.8≤|*d*_*i*_ */a_*i*_ *|<1.2), or overdominant (|*d*_*i*_ */a_*i*_ *|≥1.2).

## Results

### Linkage map construction

**Fig 1:**
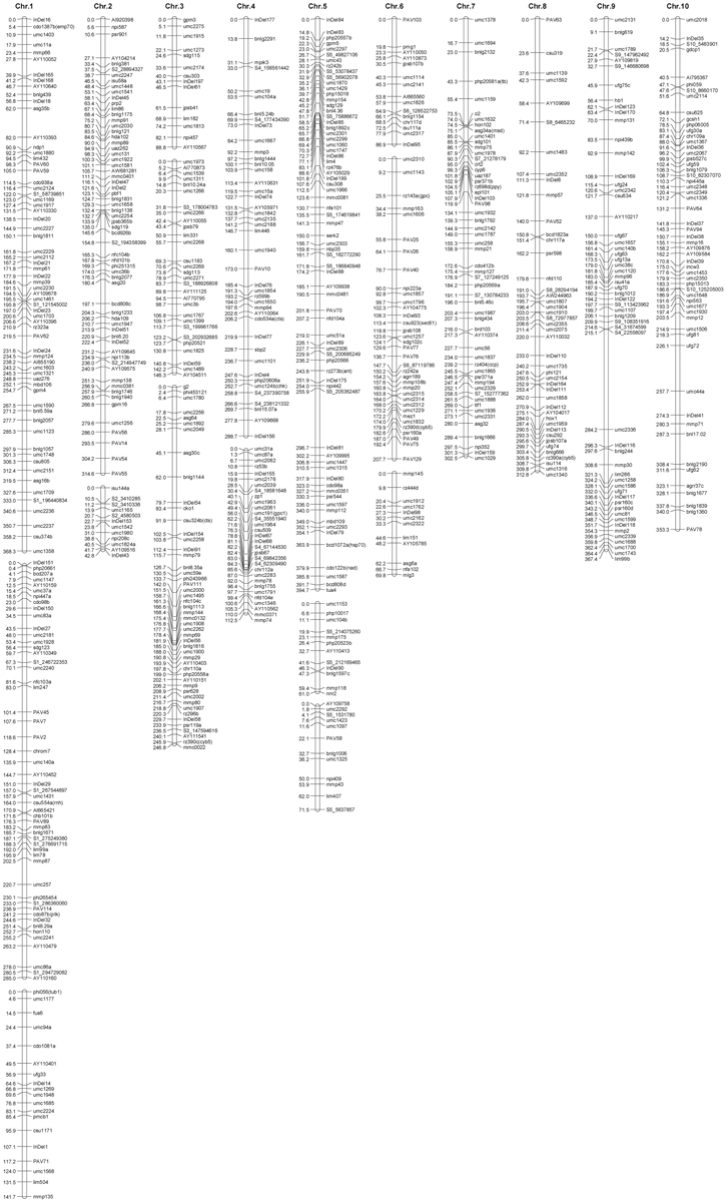
Linkage map generated from 302 individuals. Each chromosome is comprised of one or more LGs and each LG is represented by one bar. The bars of each chromosome were aligned vertically and not according to the order of chromosome framework. The numbers on the left of the bar represent the genetic position of markers as evaluated by the distance function of Kosambi in cM. The names on the right side of the bar indicate the markers.

**Fig 2:**
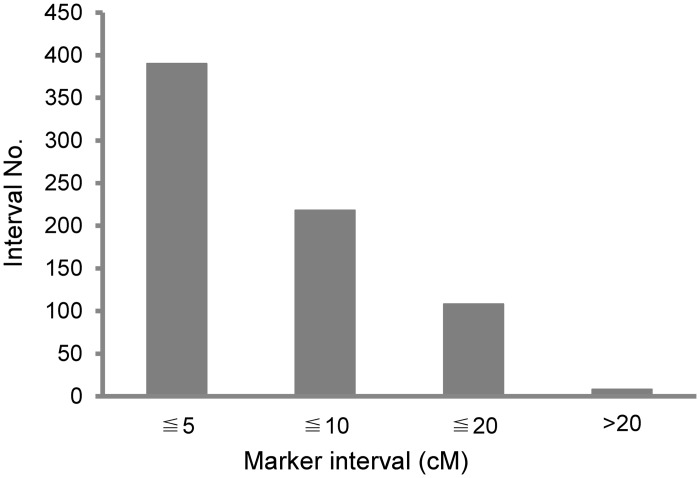
Overview of genetic distance between two adjacent markers on the linkage groups.

Linkage map construction was carried out according to the procedure of MSTMap based on the 302 IBM individuals. The complete linkage map was comprised of 77 InDels, 32 PAVs, 61 SNPs, and the remaining 574 other public markers. In total, 744 markers were assigned to 20 linkage groups (LGs). Among these LGs, there were four with 12 markers each and 8 with 14 to 71 markers each (Fig 1). The average size of the 20 LGs was 213.2 centiMorgan (cM), ranging from 42.8 cM to 394.7 cM. The linkage map covered all 10 maize chromosomes and spanned 4263.1 cM of the genome with an average interval distance of 5.7 cM between markers (Fig 1). The minimum distance between markers was 0.2 cM, while the maximum marker interval was 69.1 cM. Among 724 marker intervals, the majority of inter-marker distances were no more than 10 cM (608≤10 cM marker intervals including 390≤5cM), 108 marker intervals were between 10 and 20 cM, and only 8 marker intervals were above 20 cM length (Fig 2). The maximum number of markers appeared on chromosome 1 (136 markers) and the minimum number appeared on chromosome 8 (44 markers). However, a few large distances still existed between adjacent markers (≥20cM); this large gap is likely due to the lack of recombination among the close markers. In addition, the large gap might cause several LGs not linked to each other although they were assigned on the same chromosome.

### Heterosis and population performance of KSRTs

**Fig 3:**
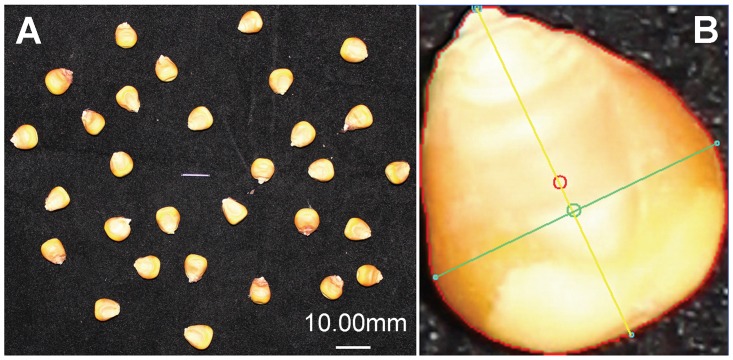
Images of kernel shape related traits detected by *SmartGrain*. (A) The loading image for *SmartGrain*. The gray wire in the center of the image is used as the reference length. (B) KSRTs recognized by *SmartGrain*. The red outline can be used for calculating PL and KA (areas within the red line); The yellow and green lines indicate the KL and KW, respectively, and can also be used for calculating LWR and CS; The small red circle and green circle represent the center of gravity and the intersection of length and width, respectively, as evaluated by *SmartGrain*. These values are not included in our analysis.

KSRTs of all the populations in our study were evaluated by *SmartGrain* (Fig 3A). The image shows that the various outlines of seed traits could be recognized clearly (Fig 3B) and it indicates that KSRTs have been measured accurately. The mean values and heterosis of KL, KW, LWR, PL, KA, and CS for basic populations are listed in Table 1. For parental lines B73 and Mo17, the performance of KW, LWR, and KA differed significantly and the mean values of KW and KA in B73 were significantly lower than Mo17. Except for CS, the seed trait values of F_1_ were significantly higher than the MP values. When comparing F_1_ with two parental lines, the mean values were greater than both of parents. This indicated that most of the KSRTs exhibited positive dominance, even overdominance effects. Only CS exhibited negative dominance effect. Heterosis of KSRTs was estimated via MPH. KA exhibited the highest level of MPH (>40%) among these seed traits, MPH values of KL and PL were >20%, and MPH values of KW and LWR were >10%. However, CS expressed very low levels of MPH.

**Table 1:** Mean values and heterosis based on the three blocks.

Generation	KL (mm)	KW (mm)	LWR	PL (mm)	KA (mm^2^)	CS
B73	8.38±0.35	6.60±0.42	1.23±0.03	25.48±1.41	41.25±2.88	0.81±0.01
Mo17^a^	8.96±0.65	7.87±0.27**	1.14±0.04*	28.06±1.84	50.57±5.92*	0.81±0.01
MP	8.51±0.43	7.23±0.35	1.18±0.03	26.77±1.53	45.91±4.17	0.81±0.01
F_1_ ^b^	10.99±0.47**	8.08±0.43*	1.39±0.02**	32.40±1.52**	67.21±6.70**	0.8O±0.01
MPH (%)	26.8±2.53	14.54±3.10	17.97±3.83	21.11±2.36	46.33±4.64	-1.23±1.97

* *P*≤0.05,

** *P*≤0.01.

^a^ Comparison between B73 and Mo17 using *t* test.

^b^ Comparison between MP and F1 using *t* test.

The maximum, minimum, and mean values of four populations across three blocks are shown in Table 2. The performance of all seed traits for RILs (Table 2) was almost at the same level with the MP of the parents (Table 1). This stable mean value demonstrates that there was no interference in the artificial selection during the continuous self-pollination program for creating the RIL population from F_1_. Meanwhile, the maximum and minimum values of RIL traits were more extreme than those of the high value parent and low value parent, respectively. Thus, the RILs displayed extensive variation beyond the parents. When we compare the performance of RILs (Table 2) and F_1_ (Table 1), F_1_ values fall into the range between the maximum and minimum values of RILs for all KSRTs. This indicates the moderate MPH of KSRTs.

**Table 2:** Maximum, minimum, and mean values of RIL and TC populations based on three blocks.

Population	Number		KL (mm)	KW (mm)	LWR	PL (mm)	KA (mm^2^)	CS
		max	11.99	9.07	1.63	34.89	75.55	0.86
RIL	82	min	7.47	5.74	1.10	23.51	35.78	0.74
		mean	9.40±0.90	7.57±0.61	1.25±0.10	28.64±2.41	53.09±8.43	0.81±0.02
		max	12.00	9.19	1.56	35.34	78.94	0.85
TC(B)	82	min	7.98	6.27	1.09	24.51	38.94	0.72
		mean	10.01±0.75	7.71±0.55	1.31±0.09	30.05±2.05	57.66±7.48	0.80±0.02
		max	12.31	9.63	1.59	36.12	83.24	0.85
TC(M)^a^	82	min	8.71	6.98	1.09	26.88	46.74	0.74
		mean	10.47±0.60**	8.46±0.50**	1.27±0.09**	31.97±1.69**	65.87±6.68**	0.81±0.02**
		max	12.28	9.76	1.55	37.07	88.93	0.85
TC(F)^b^	82	min	7.84	5.99	1.07	24.93	39.84	0.73
		mean	10.34±0.75	8.03±0.58	1.27±0.09**	31.15±2.11	62.04±7.96	0.80±0.02

** *P*≤0.01.

^a^ Comparison between TC(B) and TC(M) using *t* test.

^b^ Comparison between TC(F) and the mean of TC(B) and TC(M) using *t* test.

With respect to TC progenies, mean values of TC(M) were significantly higher than TC(B) for KL, KW, PL, and KA. On the contrary, the values were significantly lower for LWR (Table 2) which is consistent with the observation of parental Mo17 to B73 and confirms the contribution of beneficial alleles donated from Mo17 and B73, respectively, to the former traits and the latter trait. As expected, both of the means of crossed heterozygous progenies from TC(B) and TC(M) populations exceeded significantly any homozygous parent of RIL, B73, and Mo17 populations for most of KSRTs (data not shown). This indicates the prevalence of heterosis. Additionally, only LWR exhibited significant difference between the mean of TC(F) and the mean of TC(B) and TC(M). Furthermore, the mean of TC(F) trended higher than the homozygous parent RIL mean and lower than the heterozygous parent F_1_ mean for most of the KSRTs. The CS of progenies did not exceed that of any parent. The phenotypic performance difference seems to be relative with heterozygosity (F_1_>TC(F)>RIL).

**Fig 4:**
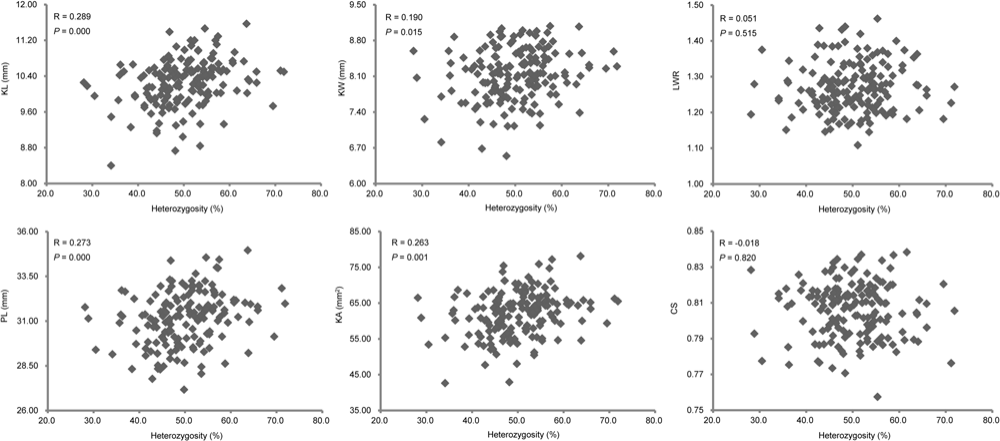
The relationship between heterozygosity and KSRTs based on TC(B) and TC(M) populations.

To further explore the relationship between the heterozygosity and the KSRTs, we investigated the correlations between the heterozygous levels of TC(B) and TC(M) individuals (based on the homozygous proportion of RILs inherited from B73 and Mo17) and their KSRTs (S3 Table). Four out of the six traits exhibited significant positive correlation with heterozygosity (Fig 4). However, the Pearson’s R values showed that the correlations between the heterozygosity and the KSRTs were not strong. In the meanwhile, the correlations among KSRTs and between KSRTs and HKW were detected (Table 3). Among KSRTs, KL was not correlated with other KSRTs, whereas KW was statistically significant correlated with the KSRTs except KL. The strongest correlation was found between PL and KA in which the R values was up to 0.964. Four KSRTs were shown to be significant positive correlation with HKW, and significant negative correlation was found between LWR and HKW. The correlation between KL and HKW was not existed according to the corresponding *P* value.

**Table 3:** The Pearson’s R correlation values among KSRTs, and between KSRTs and HKW.

Trait	R value					
	KW(mm)	LWR	PL(mm)	KA(mm^2^)	CS	HKW(g)
KL(mm)	0.042	-0.023	0.042	0.028	-0.036	0.013
KW(mm)		-0.399**	0.816**	0.889**	0.252**	0.475**
LWR			0.036	-0.075*	-0.404**	-0.155**
PL(mm)				0.964**	-0.128**	0.403**
KA(mm^2^)					0.044	0.442**
CS						0.283**

* *P*≤0.05,

** *P*≤0.01.

### Identification of QTLs affecting kernel shape

QTL analysis was carried out based on *Z1*, *Z2*, and *Z3* of three blocks. The putative QTL was declared significant when *P*≤0.001 and *R* ^*2*^>5% or when *P*≤0.001 and LOD>3.0 (0<*R* ^*2*^≤5%). In total, 28 QTLs for KSRTs were detected in three *Z* transformations with some QTLs related to different traits located within the same QTL regions (Table 4). The contribution of a single QTL to total phenotypic variation ranged from 2.1% to 32.9% with 18 QTLs >5% (Table 4). The majority of LOD values exceeded the 3.0 with a maximum of 9.3. However, the LOD of two QTLs did not achieve the threshold with a minimum of 2.4 (data not shown).

**Table 4:** QTLs for KSRTs detected in *Z1*, *Z2*, and *Z3*.

KSRTs	QTL	Chr.	Marker interval		*Z1*			*Z2*		Gene action^a^		*Z3*	
				LOD	*a*_*i*_ *	*R* ^*2*^ (%)	LOD	*d*_*i*_ *	*R* ^*2*^ (%)		LOD	*da*_*i*_	*R* ^*2*^(%)
KL	*qKL1a*	1	ufg33-InDel14	2.78	-0.16	12.3				A			
	*qKL2a*	2	S2_28894327-umc2247				4.94	0.30	4.0	OD			
	*qKL5a* ^b^	5	umc1447-umc1315				5.66	0.31	4.3	OD			
	*qKL5b*	5	AY109758-umc2292								2.43	0.51	18.6
	*qKL6a*	6	AI665560-umc1826				4.97	0.27	3.3	OD			
	*qKL8a*	8	psr598-nfd110				3.80	-0.35	5.5	OD			
	*qKL9a*	9	InDel169-ufg24				5.54	0.30	4.0	OD			
KW	*qKW4a*	4	InDel73-umc1667								6.23	-0.36	15.7
LWR	*qLWR1a*	1	bnlg439-InDel18	5.76	0.02	6.0				A			
	*qLWR2a*	2	umc1658-bnlg1138	8.99	0.03	13.4				A			
	*qLWR3a*	3	mmp80-umc1907	6.13	0.02	6.0				A			
	*qLWR5a*	5	csu308-umc1966	5.01	0.02	6.0				A			
	*qLWR6a*	6	umc1114-umc2141				3.11	0.03	7.7	OD			
	*qLWR6b*	6	mmp163-umc1606				6.49	0.04	13.6	OD			
	*qLWR8a*	8	umc2355-umc2075	9.26	-0.02	6.0				A			
	*qLWR9a*	9	umc2131-bnlg619				3.70	0.03	7.7	OD			
	*qLWR10a*	10	php06005-ufg30a	5.41	-0.02	6.0				A			
PL	*qPL1a* ^c^	1	umc1948-umc1685	5.58	-0.57	29.0				A			
	*qPL5a* ^b^	5	umc1447-umc1315				2.97	0.68	6.4	OD			
	*qPL5b* ^d^	5	InDel175-npi442								7.16	-1.06	7.7
	*qPL9a* ^e^	9	umc2342-csu634				4.97	0.99	13.6	OD			
	*qPL10a* ^f^	10	ufg81-ufg72								6.92	1.23	10.3
KA	*qAS1a* ^c^	1	umc1948-umc1685	3.32	-1.67	20.1				A			
	*qAS5a*	5	umc1315-InDel80				3.47	2.74	4.8	OD			
	*qAS5b* ^d^	5	InDel175-npi442								3.72	-5.24	2.7
	*qAS9a* ^e^	9	umc2342-csu634				5.14	3.50	7.8	OD			
	*qAS10a* ^f^	10	ufg81-ufg72								3.30	5.39	2.1
CS	*qCS6a*	6	PAV40-npi223a				3.36	-0.01	32.9	OD			

^a^ Degree of dominance: additive (A, |*d*_*i*_ */a_*i*_ *|<0.2), partially dominant (PD, 0.2≤|*d*_*i*_ */a_*i*_ *|<0.8), dominant (D, 0.8≤|*d*_*i*_ */a_*i*_ *|<1.2), and overdominant (OD, |*d*_*i*_ */a_*i*_ *|≥1.2).

^b, c, d, e, f^ indicates marker intervals that were mapped to more than one QTL.

Seven QTLs were detected for KL: five in *Z2* and one in both *Z1* and *Z3* (Table 4). These QTLs were mapped on six chromosomes. Despite five dominant QTLs detected in *Z2* that were all characterized by overdominance, these QTLs could not be considered as major QTLs due to low *R* ^*2*^ values (3.3%-5.5%). However, these dominant QTLs found in *Z2* explained 21.0% of total phenotypic variance. The negative additive QTL detected in *Z1* located within the marker region of ufg33-InDel14 on chromosome 1 accounted for 12.3% of the phenotypic variation which indicates that an allele from the longer kernel parent Mo17 contributed to improving kernel length. In *Z3*, one epistasis QTL was examined which explained 18.6% of the phenotypic variation.

There was only one QTL detected for KW (Table 4): *qKW4a* was identified in *Z3*, mapped on chromosome 4, accounted for 15.7% of the phenotypic variation, and displayed an epistasis effect. It is suggested that the dominance × additive epistatic interaction might have an effect on kernel width.

Nine QTLs were identified for LWR: six in *Z1* and three in *Z2* (Table 4). These QTLs were dispersed on chromosomes 1, 2, 3, 5, 8, and 10 (one QTL per chromosome for *Z1*) as well as on 6 and 9 (for *Z2*). The QTLs in *Z1* exhibited additive effects and QTLs in *Z2* displayed overdominant effects. Among additive QTLs, four were positive and two were negative, indicating that alleles from both large LWR parent B73 and small LWR parent Mo17 were beneficial for increasing LWR. The LOD of additive QTLs were all greater than 5.0, and their contribution ranged from 6.0% to 13.4%. Two of three dominant QTLs were located on chromosome 6 and the other was on chromosome 9. They were all classified as positive overdominance, explaining individually between 7.7% and 13.6% of total variation. QTLs detected in *Z1* accounted for 43.2% of total phenotypic variation due to additive effects, whereas QTLs identified in *Z2* accounted for 29.0% of total phenotypic variation due to dominance effects.

Five QTLs were found for PL: one in *Z1* and two in both *Z2* and *Z3* (Table 4). The QTL found in *Z1* contributed 28.6% to total phenotypic variation and exhibited a negative additive effect. This indicates that an allele from high PL parent Mo17 contributed to improving PL. Two QTLs identified in *Z2* were located on chromosomes 5 and 9, respectively, and both showed positive dominant effects with overdominance classification. The simultaneous fit of two overdominant QTLs accounted for 20.0% of total variation (6.4% and 13.6% each). Moreover, *qPL5a* flanked with umc1447 and umc1315 shared a genomic region with overdominant *qKL5a* detected in *Z2* for KL. For *Z3*, two QTLs found in chromosomes 5 and 10, respectively, exhibited opposite epistatic effects and explained 7.3% and 10.7% of total variation each.

Likewise, five QTLs were detected for KA: one in *Z1* and two in both *Z2* and *Z3* (Table 4). The mapped genetic position and the orientation of genetic effects of QTLs for KA were almost exactly the same as the five QTLs for PL, except for *qAS5a* and *qPL5a* with two mapping positions closely linked. In addition, the contribution to total variation explained by individual QTLs of KA was all lower than those of PL.

For CS, only one QTL were found. The QTL detected in *Z2* was mapped on chromosome 6. This overdominant QTL accounted for 32.9% of total variation which was the maximum contribution of all individual QTLs detected.

### Analysis of digenic interaction affecting KSRTs

Digenic epistasis for QTLs detected in *Z1* and *Z2* was determined based on an analysis of marker pairs over the whole genome. The epistatic effects detected in *Z1* and *Z2* are referred to as additive × additive and dominance × dominance interaction effects, respectively. In *Z1*, the interaction genetic regions were found for all traits, whereas, only three traits in *Z2* (KL, LWR, and CS) were found to possess digenic interaction. Table 5 shows the digenic epistasis detected in *Z1* and *Z2*, excluding those with *R* ^*2*^_*ij*_ of zero. Totally, there were 28 pairs of digenic epistasis detected for all traits in *Z1* and *Z2*. The number of the digenic interaction pairs for each KSRT ranged from one for KA to nine for LWR.

**Table 5:** The genetic effect and contribution of epistatic QTL for KSRTs detected in *Z1* and *Z2*.

KSRTs		Chr.	Marker interval	Chr.	Marker interval	LOD	*a*_*i*_ ^*a*^	*a*_*j*_ ^*a*^	*aa*_*ij*_ ^*a*^	*R* ^*2*^_*i*_(%)^b^	*R* ^*2*^_*j*_(%)^b^	*R* ^*2*^_*ij*_(%)^b^	*R* ^*2*^(%)^b^
KL	*Z1*	4	umc2176-umc2039^c^	1	ufg33-InDel14^d^	3.05	0.02	-0.13**	-0.12	0.3	12.1	10.3	10.3
	*Z2*	5	AY109995-umc1447	5	umc104b-S5_214075260	5.08	0.31**	0.13	-0.14	8.7	1.5	1.8	11.7
		7	rz404-umc1865	4	bnl10.05-umc158	4.30	-0.10	-0.13	0.29**	0.9	1.5	7.6	
		8	psr598-nfd110	4	umc2176-umc2039^c^	3.97	-0.44**	0.05	0.16	17.5	0.2	2.3	
KW	*Z1*	2	npi587-psr901	1	ufg33-InDel14^d^	10.87	-0.01	-0.01	-0.12**	0.1	0.1	11.8	78.8
		2	AY109575-mmp138	5	S5_53078437-S5_56902078	8.45	-0.04	-0.06	-0.18**	1.3	3.0	26.5	
		3	umc59e-phi243966	1	S1_294729082-AY110160	4.08	0.00	0.00	0.09**	0.0	0.0	6.6	
		3	AY111541-rz390c	5	umc1747-InDel86	4.61	-0.02	-0.01	-0.09**	0.3	0.1	6.6	
		3	jpsb79-lim331	7	umc1787-umc258	7.47	0.00	0.00	0.11**	0.0	0.0	9.9	
		4	InDel67-InDel68	5	php15018-mmp154	5.23	0.01	-0.01	0.09**	0.1	0.1	6.6	
		6	S6_128522753-bnlg1154	5	InDel24-mmp124	2.93	-0.01	0.01	-0.07**	0.1	0.1	4.0	
		6	mmp16-AY109876	5	umc43-rz242b	4.32	0.01	0.01	0.09**	0.1	0.1	6.6	
LWR	*Z1*	1	csu374b-umc1358	5	php20566-S5_200685249	5.83	0.00	0.00	-0.02**	0.0	0.0	6.3	30.1
		3	umc1907-rz296b	4	umc2176-umc2039^c^	5.41	0.02**	0.00	-0.01	6.3	0.0	1.6	
		3	umc2268-csu1183	7	sdg101-mmp75	3.65	-0.02**	0.00	-0.03**	6.3	0.0	14.2	
		3	bnlg1113-mmp144	9	ufg24-umc2342	5.09	0.00	0.00	0.02**	0.0	0.0	6.3	
		4	PAV10-InDel76	1	bnlg439-InDel18	5.61	0.00	0.02**	-0.01	0.0	6.3	1.6	
	*Z2*	3	mmp144-mmc0132	9	umc2131-bnlg619	5.09	0.00	0.04**	0.02	0.0	12.7	3.2	20.7
		6	umc1114-umc2141	4	umc2176-umc2039^c^	3.89	0.04**	0.02	0.02	12.7	3.2	3.2	
		6	mmp108b-mmp20	10	umc2114-csu625	4.14	0.00	0.00	-0.03**	0.0	0.0	7.2	
		8	umc1483-umc2352	1	AY109678-umc1461	4.46	-0.01	0.01	-0.03**	0.8	0.8	7.2	
PL	*Z1*	4	umc2176-umc2039^c^	1	umc1948-umc1685	3.98	-0.09	-0.49**	-0.24	0.7	20.9	5.0	7.8
		9	csu634-AY110217	4	umc2176-umc2039^c^	4.86	0.95**	-0.25	0.31	25.8	1.8	2.8	
KA	*Z1*	8	S8_72977857-umc2355	1	umc1403-umc11a	4.91	0.10	-0.07	-3.21**	0.0	0.0	17.0	17.0
CS	*Z1*	2	InDel45-prp2	10	phi059-S10_8660170	4.95	0.00	0.00	-0.01**	0.0	0.0	31.1	62.2
		3	php20521-umc1825	2	mmc0401-InDel47	6.41	0.00	0.00	-0.01**	0.0	0.0	31.1	
	*Z2*	2	umc1658-bnlg1138	5	nfd104a-umc51a	4.20	0.00	0.00	0.01**	0.0	0.0	27.7	55.3
		4	bnlg1444-bnl10.05	5	mmp154-sdg129	6.90	0.00	0.00	-0.01**	0.0	0.0	27.7	

** *P*≤0.01.

^a^ *a*_*i*_ and *a*_*j*_ represent the main effect of the loci *i* and *j*, and *a*_*ij*_ represents the epistatic effect between loci *i* and *j*.

^b^ *R* ^*2*^_*i*_, *R* ^*2*^_*j*_, *R* ^*2*^_*ij*_, and *R* ^*2*^ represent the genetic contribution via the percentage of the total variation explained by the *a*_*i*_, *a*_*j*_, *a*_*ij*_ and the total interactions for KSRTs respectively.

^c, d^ indicate marker intervals mapped to more than one QTL.

The majority of interaction effects detected in the KSRTs were determined to be significant. The digenic epistatic effects were all significant for KW, KA, and CS. However, there was no significant epistatic interaction detected for PL. In Table 5, most of the single genetic regions which displayed no or a small contribution to phenotypic variation could account for large proportions of total variation when two genetic regions interacted. For example, no single locus detected in CS could be shown as contributing to phenotypic variation. However, two locus interactions could both explain ~30% of total variation. There were also some cases where the percentage of total variation due to digenic interaction was obviously lower than that due to a single locus. For example, two genetic regions detected in *Z1* for PL accounted for 25.8% and 1.8% of total variation, respectively, but the contribution to total variation explained by their digenic interaction was 2.8%. In addition, the proportion of total variation explained by epistatic interactions detected in *Z1* and *Z2* ranged from 1.6% to 31.1%. In *Z1*, the maximum proportion of variation explained by all additive × additive interactions was 78.8% for KW. This is consistent with the observation that epistatic QTL was detected in *Z3* without any significant additive or dominant QTLs detected in *Z1* or *Z2* for KW. This suggests that epistasis interaction may play an essential role in determining maize KW. In *Z2*, the highest total *R* ^*2*^ was 55.3% for CS and resulted from a dominance × dominance interaction.

The majority of genetic regions detected in digenic epistasis analysis exhibited interaction with one another genetic region. However, some digenic epistatic regions were found to have simultaneous interaction with multiple regions. The genetic regions flanked by umc2176 and umc2039 were discovered to have epistatic interactions with various loci for the three traits KL, LWR, and PL. These involved both additive × additive and dominance × dominance interactions which indicate that kernel shape may be regulated by digenic epistasis at this region. Likewise, additive × additive epistatic interactions were found between the genetic region enclosed by ufg33 and InDel14 and two other loci for both KL and KW. Furthermore, this common interactive region was found to be shared with an additive QTL for KL. Most of loci exhibited main-effect and digenic epistatic effect contributing to the same trait (e.g., psr598-nfd110 for KL and bnlg439-InDel18, umc1114-umc2141, and umc2131-bnlg619 for LWR). We also found that the genetic region of umc1658-bnlg1138 on chromosome 2 mapped with an additive QTL for LWR and contributed to CS via a dominance × dominance interaction with the genetic region on chromosome 5. However, it should be noted that the majority of interactive regions were not mapped with main-effect QTLs. Moreover, no two regions showing digenic interaction were found to both co-locate with main-effect QTLs. In addition, the genetic region harboring an epistatic QTL detected in *Z3* was not shown to interact with other genetic regions.

## Discussion

### Heterosis, mode of gene action for KSRTs, and their utilization in maize breeding

Heterosis has been widely used in maize breeding for improving production [3–5]. Several key traits attracted breeders have been focused on dissecting their genetic basis [50,51]. Among traits with various heterotic levels, KSRTs are considered as important components involved in grain yield. In maize breeding, high length and low width kernels are the preference of breeders because slim kernels provide growing space for closely packed kernels and long kernels increase the grain yield if ear diameter remains unchanged. Therefore, the long KL type is expected to be the initial kernel shape trait in breeding programs.

In present study, the highest MPH was 46.33% for KA which was followed by KL (26.8%). It seems that the KSRTs exhibited modest heterosis levels. For traits showing strong heterosis, their MPH level might be more than 100%. For example, MPH of seedling dry weight and number of kernels per plant were found to exceed 100% and even that of grain yield could reach 239% when maize inbred line B73 was crossed with H99 [41]. It is not surprising that different traits expressed various heterosis levels due to the cross between particular maize inbred lines. According to the interpretation of MPH defined by Melchinger et al. [46], MPH can be expressed as the sum of augmented dominance QTL effects. In the formula, the augmented dominance effect is denoted as the dominance effect minus half the sum of its additive × additive interactions. Thus, the dominance and additive × additive effects both contribute to MPH. For most of the KSRTs, augmented dominance QTLs were detected in *Z2*, except for KW which only displayed epistasis. The mode of gene action of augmented dominance QTLs were all overdominance, suggesting that overdominance or epistasis might contribute to these QTLs. To improve MPH for these traits, the sum of dominance effects of QTLs should increase to a greater extent than the sum of additive × additive interactions during breeding.

The augmented additive QTLs were also identified in *Z1* for four out of six KSRTs (excluding KW and CS). The augmented additive effect at a QTL was considered as the net contribution of the QTL to the parental difference in the presence of epistasis [46]. The contribution to phenotypic variation explained by an individual QTL for KL was 12.3%, indicating that selection based on the augmented additive locus should be enhanced in the process of maize breeding. For all QTLs identified, the total variation explained by individual QTLs ranged from 2.1% to 32.9%. If marker-assisted selection (MAS) was used for KSRTs, the QTLs with minor contributions should not be abandoned because the selection for beneficial genotypes with more loci would improve the target phenotype value. However, MAS for epistatic QTLs should be cautious due to the difficulties in the prediction of epistatic effects.

In our study, the change in phenotypic values of KSRTs can be attributed to B73 and Mo17, implying that alleles from both parents contribute to KSRTs. For the complex traits, it is not difficult to understand that positive effects for different components might be provided by any parent which leads to the observation that the alleles from the two parents were both in favor of the whole complex trait. This has been observed in other studies. For example, the complex trait grain yield gained favorable alleles from both Zong3 and 87–1 parents [18]. Taken together, the occurrence of additive, dominant, and epistatic QTLs for components of KSRTs indicates the genetic complexity of KSRTs. To gain a better understanding of the genetic basis of complex maize KSRTs for their further utilization, the favorable genes should be isolated and characterized.

### Comparison of QTLs for KSRTs

RIL-based genetic design has been proven providing the highest power for QTL detection than other populations [52]. In our study, 28 QTLs were found for six KSRTs using RIL-based TTC populations. Among these QTLs, 10 QTLs involved in three traits were found in five common genomic regions with each region mapped with two QTLs (Table 4). The QTL region flanked with umc1447 and umc1315 for KL were identified as a QTL for PL with the same mode of gene action. Furthermore, this region was found to be closely linked with a QTL surrounded by umc1315 and InDel80 for KA. Likewise, four QTL regions for PL were also determined to be QTLs for KA with the same types of gene action. Thus, QTLs for PL seem identical to those for KA. One possible explanation for the high consistency of QTLs mapped for these two traits is that KA has the close correlation with PL (Table 3). Moreover, several main-effect QTL regions for one trait expressed interaction with genomic regions for the same trait or another trait. Therefore, some QTL regions might play pleiotropic roles for different traits. On the other hand, traits that are closely linked might also lead to the co-localization for different traits.

To evaluate the consistency of QTLs for KSRTs, we collected the QTLs for KL and KW reported in recent studies [17,36]. The comparison results revealed the poor congruency among these QTLs. No QTLs were located on the same chromosomal bins. The inconsistency of QTLs for the same traits has been reported in previous studies [53,54]. The main cause of the discrepancy is usually attributed to genetic background differences which result from utilizing various mapping populations.

### Interpretation of digenic interaction

In addition to main effect, epitasis was also exhibited large effect on complex traits in maize, especially grain yield [55,56]. The types of digenic interactions including additive × additive, additive × dominance and dominance × dominance have been shown to exist in different proportions for the target traits. Additive × additive is usually more common than the other interactive types. For instance, additive × additive epistatic interaction was the major interactive type among digenic interactions detected in maize grain yield and yield components using F_2:3_ populations [18]. In another study on maize kernel related traits, additive × additive interaction made up the highest proportion among all interactive types and displayed the large digenic epistatic effects on KL and kernel thickness [35].

In our present work, the proportion of 67.9% for additive × additive interactions detected in *Z1* versus 32.1% for dominance × dominance interactions detected in *Z2* illustrates the importance of additive × additive digenic interactions for KSRTs. For KL, the contribution to total variation explained by individual additive × additive interactions was higher than that by dominance × dominance interaction. Moreover, for KW, the epistasis detected in *Z3* was all attributed to eight additive × additive interactions with a total *R* ^*2*^_*ij*_ of 78.8%. Therefore, additive × additive epistatic interactions between two loci may be the important genetic element for KL and KW. On the other hand, only one additive × additive interaction was detected for KA which had the highest MPH. This result demonstrates that reduced additive × additive interaction can increase the MPH for the trait according to the definition of Melchinger et al. [46]. Furthermore, there was no dominance × dominance digenic interaction found for KW, PL, or KA. This suggests that dominance × dominance digenic interactions might not be important for heterosis of these traits.

However, most of the interactive genomic regions were not mapped with main-effect QTLs. The majority of the single loci were not significant for the KSRTs but exhibited significant interactive effects. This finding is in agreement with a previous study which reported that 74.9% of significant epistatic interactions occurred between loci not linked with any QTL and that only a few interactive loci were coupled with main-effect QTLs [18]. These results indicate that some main-effect QTL regions could influence the genetic background of corresponding KSRTs.

It should be noted that high-order epistasis might influence on KSRTs even though proper evaluation could not be conducted due to the detection methods of our study. All inferences on epistatic interaction should be cautious when the size of mapping population is limited [57].

## Supporting Information

S1 Table The primer list of insertion/deletion (InDel) and presence/absence variations (PAV) markers anchored in the linkage groups (LGs). (XLSX)

S2 Table The genotyping information of intermated B73 × Mo17 (IBM) recombinant inbred lines (RILs) used for genetic likage group construction. "A" and "B" represent the genotype of B73 and Mo17 respectively, and "-" represents the missing genotype. "S1_10096204" represents the single nucleotide polymorphism (SNP) occurred in the position 10096204 on Chromosome 1 of B73 reference genome (V2). The naming format is applicable for other SNPs.
(XLSX)

S3 Table The homozygous proportion of intermated B73 × Mo17 (IBM) individuals and heterozygosity of TC(B) and TC(M) populations. (XLSX)
